# Novel Quantification of Extracellular Vesicles with Unaltered Surface Membranes Using an Internalized Oligonucleotide Tracer and Applied Pharmacokinetic Multiple Compartment Modeling

**DOI:** 10.1007/s11095-021-03102-z

**Published:** 2021-10-20

**Authors:** Thomas De Luca, Robert E. Stratford, Madison E. Edwards, Christina R. Ferreira, Eric A. Benson

**Affiliations:** 1 Division of Nephrology, Department of Medicine, Indiana University School of Medicine, Indianapolis, Indiana 46202 USA; 2 Division of Clinical Pharmacology, Department of Medicine, Indiana University School of Medicine, Indianapolis, Indiana 46202 USA; 3 Department of Chemistry, Purdue University, West Lafayette, Indiana 47907 USA; 4 Bindley Bioscience Center, Purdue University, West Lafayette, Indiana 47907 USA; 5 Eli Lilly and Company, Indianapolis, Indiana 46225 USA

**Keywords:** droplet digital PCR (ddPCR), extracellular vesicles, exosomes, pharmacokinetics, tracer miRNA

## Abstract

**Purpose:**

We developed an accessible method for labeling small extracellular vesicles (sEVs) without disrupting endogenous ligands. Using labeled sEVs administered to conscious rats, we developed a multiple compartment pharmacokinetic model to identify potential differences in the disposition of sEVs from three different cell types.

**Methods:**

Crude sEVs were labeled with a non-homologous oligonucleotide and isolated from cell culture media using a commercial reagent. Jugular vein catheters were used to introduce EVs to conscious rats (n = 30) and to collect blood samples. Digital PCR was leveraged to allow for quantification over a wide dynamic range. Non-linear mixed effects analysis with first order conditional estimation – extended least squares (FOCE ELS) was used to estimate population-level parameters with associated intra-animal variability.

**Results:**

86.5% ± 1.5% (mean ± S.E.) of EV particles were in the 45–195 nm size range and demonstrated protein and lipid markers of endosomal origin. Incorporated oligonucleotide was stable in blood and detectable over five half-lives. Data were best described by a three-compartment model with one elimination from the central compartment. We performed an observation-based simulated posterior predictive evaluation with prediction-corrected visual predictive check. Covariate and bootstrap analyses identified cell type having an influence on peripheral volumes (V2 and V3) and clearance (Cl3).

**Conclusions:**

Our method relies upon established laboratory techniques, can be tailored to a variety of biological questions regarding the pharmacokinetic disposition of extracellular vesicles, and will provide a complementary approach for the of study EV ligand-receptor interactions in the context of EV uptake and targeted therapeutics.

**Supplementary Information:**

The online version contains supplementary material available at 10.1007/s11095-021-03102-z.

## Introduction

Extracellular vesicles (EV) can be used to improve medical treatments if properly understood (1, 2). Chief among the EV subtypes that have captured the interest of clinical researchers are exosomes, which are small (< 200 nm) EVs that begin as the intraluminal vesicles of the late stage endosome, where they are loaded with active biological molecules such as microRNAs (miRNA), mRNA, and proteins (3). Once secreted, they transport these contents to other nearby cells or to distant tissues via the blood circulation. Targeted distribution of these vesicles is governed by surface markers, the composition of which is dependent on the originating cell (4–6). Since EVs are continually secreted by virtually every eukaryotic cell, it is broadly accepted that the composition of any individual vesicle reflects the status of its originating cell at a particular moment in time. This dynamic heterogeneity in blood-circulating EVs makes the study of EV kinetics difficult (7, 8).

In order to quantitatively decipher the complexity of circulating EVs, there is a need for an easily applicable, reproducible method for determining the kinetic parameters of EVs from known origins (2). Due to the inherent difficulty of studying EV transport and distribution in humans, preclinical *in vivo* animal models are used. Existing studies of circulating EV kinetics are limited and have involved the development of membrane-associated labels and companion detection methods. The use of luciferase or radiolabels anchored to exogenously expressed transmembrane proteins (4, 9) provide exceptional kinetic information for the evaluation of engineered targeted therapeutics, but it is not ideal for the study of unmodified EVs. To arrive at a better understanding of how endogenous EV composition affects kinetics, we measured the kinetics of EVs with unmodified surface membranes.

We sought to develop an accessible and scalable approach that: 1) allows labeling of EVs without membrane surface modification, 2) provides reproducible and quantitative measurements of kinetic parameters, and 3) fits within established workflows for the computational modeling of kinetics. Here we describe a method to label the contents of EVs released from cultured cell lines and measure the kinetics of labeled EVs intravenously administered to animals. We applied this method to test a hypothesis that EVs from different non-cancer cell lines, collected and isolated in the same manner, will exhibit different kinetics *in vivo*. Labeled EVs were isolated from the enriched media of three different species-matched cell lines and introduced into the central circulation of conscious animals. Blood from each animal was collected over time, and the plasma fractions were assayed for tracer concentrations. Using a pharmacokinetics approach, we developed kinetic models of EVs from each cell line and report significant differences in the kinetic parameters between them. A three-compartment non-linear mixed effects model best describes the data and provides evidence that dispositional properties of circulating EVs are sensitive to imparted biological characteristics unique to their source.

## Materials and Methods

### Animals and Housing Conditions

All procedures were conducted in accordance with applicable federal regulations (10, 11) and following the review and approval by the Indiana University-Purdue University Indianapolis (IUPUI) Institutional Animal Care and Use Committee (project 10,715, approved 18 June 2014, and project 11,299, approved 12 September 2017). As previously described (12), adult male Hsd:Sprague Dawley rats (*n* = 30; male; > 350 g; Envigo, Indianapolis, IN) were housed individually or in pairs under standard environmental conditions for ≥ 14 d prior to surgical manipulations. Following surgical implantation of catheters, all animals were individually housed.

### Cell culture

Clone 9 hepatocyte, RFL-6 and RMC cells were obtained from American Type Culture Collections (ATCC, Manassas, VA). Upon receipt, cells were passaged 3 times to create cryopreserved stocks. All cell lines were grown per ATCC recommended culture conditions. Specific media for each cell line are as follows: F-12 K medium (Fisher Scientific, Florence, KY) with 10% fetal bovine serum (Atlanta Biologicals, Flowery Branch, GA) for Clone 9, F-12 K medium with 20% FBS for RFL-6, and DMEM (ATCC) with 15% FBS for RMC. Cryopreservation medium was complete growth medium supplemented with 5% (v/v) DMSO. All cells were grown at 37°C, 5% CO_2_. Cells were subcultured every 48–72 h, when cell density reached 75–90%.

All cell lines were authenticated for correct species and verified free of interspecies or mycoplasma contamination by IDEXX (Westbrook, ME).

### XMc39 Plasmid Design and Validation

*Caenorhabditis elegans* miR-39-3p (Accession MI0000010, cel-miR-39-3p) stem-loop sequence was retrieved from www.miRBase.org and used to design oligonucleotides for cloning into the XMIRXpress pre-linearized cloning lentivector (SBI), according to vendor’s instructions (XMIR-c39-top: 5’-GATCCAGCTGATTTCGTCTTGGTAATAAGCTCGTCATTGAGATTATCACCGGGTGTAAATCAGCTTGC-3’, XMIR-c39-bot: 5’-CTAGGCAAGCTGATTTACACCCGGTGATAATCTCAATGACGAGCTTATTACCAAGACGAAATCAGCTG-3’). “Top” and “bottom” oligonucleotides (IDT) were diluted into a volume of 20 μL low-EDTA TE buffer with a final concentration of 1 μM each. Annealing was performed in a thermal cycler as follows. Denaturation was performed at 95°C for 2 min. Annealing was performed in 4 steps to minimize the formation of secondary structures: 1) Cooling to 63.8°C over 20 min at a 30% ramp rate, then holding the sample at 63.8°C for 10 min; 2) Cooling to 46°C over 20 min at a 30% ramp rate; 3) Cooling to 23°C at a 100% ramp rate. Annealed stem-loop oligonucleotide was mixed with pre-linearized vector and ligated with T4 DNA ligase (New England BioLabs, Ipswich, MA). Stbl3 competent *E. Coli* cells (Invitrogen) were transformed with ligated plasmid per recommended protocol. Three different volumes of transformation product were used for antibiotic selection on 10 cm plates containing sterile agar (Fisher) with Miller’s LB medium (Corning, Corning, NY) and 100 μg/mL ampicillin (Teknova, Hollister, CA), incubated overnight (37°C). Colonies were selected and placed in culture tubes with 5 mL sterile selection medium (LB broth supplemented with 100 μg/mL ampicillin), then incubated overnight at 37°C with shaking. Glycerol stocks were prepared and stored at -80°C. Sanger sequencing (Supplementary Fig. 1) was performed by ACGT, Inc. (Wheeling, IL) to validate sequence insertion. Total plasmid sequencing was performed by Massachusetts General Hospital Center for Computational & Integrative Biology (MGH CCIB) DNA Core (Cambridge, MA).

**Fig. 1:**
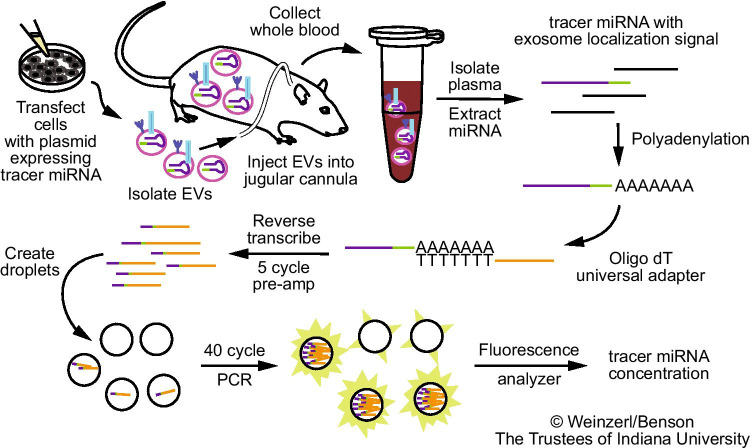
**Overview of the workflow**. Cultured cells are transfected with an expression vector encoding a non-homologous tracer miRNA stem-loop sequence with an exosome localization signal. EVs are isolated from the enriched media and administered to a conscious rat through a jugular cannula. Blood samples are collected at various time intervals, and total miRNA is extracted from the plasma. Complementary DNA is synthesized by polyadenylation and priming of the reverse transcriptase with an oligo(dT) adapter. 5-cycle PCR pre-amplification and Droplet Digital PCR are performed using primers directed to the tracer miRNA and oligo(dT) adapter. Tracer miRNA concentration is quantified using an EvaGreen fluorescence-based assay.

For scaled up plasmid production, several cultures were prepared in 1 L baffled flasks (Kimble Chase, Vineland, NJ) using a single glycerol stock, then pelleted by centrifugation. Bacterial pellets were combined and resuspended in selection medium with 25% glycerol. 1 mL aliquots were transferred to cryotubes and stored at -80°C. When needed, one aliquot was thawed to RT and added to 160 mL selection medium and incubated 20 h at 37°C with shaking. Plasmid DNA was extracted using the Qiagen HiSpeed Maxi Kit (Qiagen, Valencia, CA).

### Cell Transfection and EV Preparation

Cells were thawed and passaged at least twice (to a maximum of 5 times) before use. Cells cultured in T-75 flasks (Thermo) were grown to 70–80% confluency and transfected with 40 μg plasmid DNA using Lipofectamine 3000 (Invitrogen). After overnight incubation, cells were washed with PBS (Corning) and incubated in 10 mL cell-specific medium supplemented with 10–20% vacuum-filtered exosome-depleted FBS (SBI). After 72 h incubation, EV-enriched cell culture media was centrifuged (1,000 × g, 4°C, 10 min) in a swinging-bucket rotor to pellet residual cells and large debris. Supernatant was transferred to new conical tubes, aliquoted into 1.5 mL microcentrifuge tubes and centrifuged (10,000 × g, 4°C, 30 min) to remove large microvesicles and cell debris. The supernatants were recombined in 50 mL conical tube. Crude small EVs were isolated using Total Exosome Isolation reagent (Invitrogen). The vendor’s protocol was followed with the following additions. After overnight precipitation at 4°C, the suspension was serially pelleted by transferring aliquots to 1.5 mL microcentrifuge tubes, centrifuging (10,000 × g, 4°C, 5 min), discarding the supernatant, then adding more suspension. Approximately one 1.5 mL microcentrifuge tube was used for every T-75 flask harvested. Pellets were gently washed with PBS, softened overnight by incubation in 100 μL PBS at 4°C, and resuspended by vortexing. Resuspended EVs and residual precipitation reagent was removed by passing EVs through Exosome Spin Columns (Invitrogen). Samples were quantified by total protein content using BCA protein assays (Thermo Fisher Scientific). For western blots, samples were stored as 50 μg aliquots at -20°C. For *in vivo* dosing, samples were diluted with PBS to achieve target dose concentrations of 2 μg/ul and stored at 4°C for up to 14 d.

### RNA Extraction and cDNA Synthesis

DNA LoBind products (Eppendorf) were used where possible. RNA extractions were performed with the Qiagen miRNeasy Mini Kit (Qiagen) using 50 μL sample volumes as previously described (13, 14), the optional full speed centrifugation to remove residual buffer, and pre-heated ultrapure water (60°C) for two 30 μL elutions (60 μL total). First strand cDNA was prepared using the qScript miRNA cDNA Synthesis kit (Quantabio, Beverly, MA), using 7 μL RNA and the 20 min option for Poly(A) tailing as indicated in the vendor’s instructions. After reverse transcription (RT), samples were held at 4°C until ddPCR.

Frozen dose aliquots and plasma samples were thawed for miRNA extraction in animal-matched batches. Aliquots and samples in 1.5 mL microcentrifuge tubes were removed from storage at -80°C, and Qiazol reagent was added immediately. Samples were vortexed after thawing, incubated at RT for 5 min, then proceeded to RNA extraction.

### Droplet Digital PCR

Unless otherwise stated, all products used were from Bio-Rad. Non-targeting miRNA with Xmotif (XMIR-NT) positive control RNA oligonucleotide was purchased from SBI (Palo Alto, CA). XMc39 positive control RNA oligonucleotide was purchased from IDT (Coralville, IA): 100 nmole, UCA CCG GGU GUA AAU CAG CUU GCC UAG GAG GAG. Droplet digital PCR (ddPCR) was performed using the QX200 AutoDG ddPCR system and ddPCR Supermix for EvaGreen. Primer sequences were as follows: XMIR-NT forward primer, GAG GGC GAC TTA ACC TTA G. XMc39 forward primer, TCA CCG GGT GTA AAT CAG C; Universal reverse primer, GCA TAG ACC TGA ATG GCG GTA.

Amplification reactions of 15 μL were prepared in a DNA LoBind 96-well plate (Eppendorf) using 1.5 μL undiluted cDNA, 7.5 ul ddPCR Supermix for EvaGreen, 4.5 μL ultrapure water, and 1.5 μL 2.5 µM forward primer (final concentration 250 nM). Oligo-d(T) carryover from the RT acted as reverse primer. Samples were amplified in a thermal cycler using the following conditions: 95°C for 5 min, 5 cycles of 95°C for 30 s and 58°C for 60 s (100% ramp rate), 4°C for 5 min, 90°C for 5 min, and hold at 4°C.

We prepared ddPCR reactions according to Bio-Rad’s specifications, using 2.5 μL of the preamplification reaction and primer concentrations of 200 nM in a final volume of 25 μL. Plates were held on cold blocks to minimize activity of Taq polymerase from the preamplification reaction. Droplets were prepared using 20 μL of each supermix sample and allowed to warm to room temperature (per Bio-Rad) prior to placement in the droplet generator.

Droplets were amplified to endpoint using the following cycling conditions on a C1000 Touch thermal cycler: 95°C for 5 min, 40 cycles of 95°C for 30 s and 56°C for 60 s (default ramp rate; 2.5°C/s), 4°C for 5 min, 90°C for 5 min, and hold at 4°C. Following thermal cycling, droplets were scanned using the QX200 Droplet Reader. Analysis was performed using QuantaSoft Analysis Pro software.

### Identification of Secreted Tracer miRNA Sequence

Clone 9 cells were transfected with XMc39 lentivector and EVs were isolated. MicroRNA was extracted and cDNA was prepared. Restriction enzyme sites and 6-nucleotide 5' overhanging sequences were added to the tracer amplicon during PCR amplification using the following primers 5’ to 3’: XMc39 forward primer with BamHI, CCA CTT GGA TCC TCA CCG GGT GTA AAT CAG CTT; Universal reverse primer with EcoRI, ATC GAA GAA TTC GCA TAG ACC TGA ATG GCG GTA AG. Underlined sequences indicate BamHI (forward primer) and EcoRI (reverse primer) restriction enzyme sites. 20 μL reactions were prepared in triplicate in a 96-well plate (Applied Biosystems) as follows: 2 μL cDNA, 10 μL PowerUp SYBR Green Master Mix (Applied Biosystems), 1 μL 4 μM forward primer, 0.5 μL 10 μM reverse primer, and 6.5 μL ultrapure water (Invitrogen). Amplification was performed using the following conditions: 50°C (2 min), 95°C (10 min), 95°C (15 s), 52°C (1 min), 40 cycles of 95°C (15 s) and 61°C (1 min). All ramp rates were 1.6°C/s. An immediate melt curve analysis was performed (95°C with a 5 s hold every 0.3°C step). Triplicate PCR reactions were pooled and cleaned using a MinElute PCR Purification kit (Qiagen). Final DNA concentration was quantified using a Qubit dsDNA BR assay kit. 500 ng purified tracer cDNA (insert) and 1 μg XMc39 lentivector were separately double digested (37°C overnight) in 20 μL volumes containing 20 U BamHI-HF (NEB), 20 U EcoRI-HF (NEB), and ultrapure water. The insert digest was purified with MinElute and the plasmid digest was purified with QIAquick PCR Purification kit (Qiagen) and quantified. Ligation was performed using 20 ng digested plasmid DNA, 2.5 μL digested insert, 800 U T4 DNA Ligase (NEB), and ultrapure water in a 20 μL volume. After 10 min at 37°C, the ligase was inactivated at 65°C (10 min) prior to chilling on ice. The ligation product was introduced into competent *E. Coli* cells by heat shock and plated for antibiotic selection (see previous). Five colonies were selected and scaled up for Sanger sequencing by ACGT, Inc.

### Electron Microscopy

EVs were evaluated for morphology and contamination by the Electron Microscopy Center at Indiana University Bloomington. To prepare negative stain grid, 4 μL of sample solution was applied onto a glow-discharged 300-mesh copper grid coated with continuous carbon film (EMS, Hatfield, PA). The sample solution was left for 30 s before blotted with a piece of filter paper. The grid was washed using a 4-μL drop of milli-Q (MilliporeSigma) water and stained with 4 μL of negative stain solution composed of either 1% (w/v) uranyl acetate (EMS) with 0.5% (w/v) trehalose (MilliporeSigma) or 1% (w/v) ammonium molybdate (MilliporeSigma) with 0.5% (w/v) trehalose. Excess stain solution was removed by filter paper and the grid was allowed to air dry. Grids were imaged on a 120-kV JEM-1400Plus (JEOL USA, Peabody, MA) transmission electron microscope equipped with 4 k x 4 k OneView camera (Gatan, Pleasanton, CA).

### Nanoparticle Tracking Analysis

EV preparations were analyzed for size distribution (n = 3 biological replicates prepared on separate occasions) with dynamic light scattering using the Particle Metrix ZetaView platform (Particle Metrix, Meerbusch, Germany). Data acquisition was performed at RT using dilutions of EVs in PBS. Nanoparticle tracking analysis measurements were recorded and analyzed at 11 positions per sample with the ZetaView analysis software.

### Gel Electrophoresis and Western Blot Analysis

For whole cell lysates (WCL), adherent cells were washed in triplicate using PBS then detached by incubation in trypsin (Corning) for about 5 min at RT. Detached cells were pelleted (200 × g for 5 min at 4°C), washed 3 times with PBS, and counted using a Fuchs Rosenthal hemocytometer (Incyto, Republic of Korea). Cell pellets were lysed (1X RIPA buffer, 10 min on ice) (Cell Signaling Technology, Danvers, MA) with added protease inhibitors (Thermo) and centrifuged (14,000 × g, 10 min, 4°C). Lysate supernatants were collected and quantified by BCA assay. WCLs were stored (50 μg aliquots, -20°C) until analyzed.

EV or WCL aliquots (50 μg) were thawed in LDS sample buffer with sample reducing agent (Invitrogen), then heated at 75°C for 10 min. For probing tetraspanins, additional aliquots were prepared without the reducing agent. Denatured samples, along with Precision Plus Protein Kaleidoscope Prestained Protein Standards (Bio-Rad, Hercules, CA) and MagicMark XP Western Protein Standards (Invitrogen), were resolved on precast 4–12% Bis–Tris midi 12 + 2-well Midi protein gels (Invitrogen) at 200 V for 40 min in MES running buffer (Invitrogen), supplemented with antioxidant (Invitrogen) in the case of reduced samples. Gels were transferred to 0.45 μm PVDF membranes (MilliporeSigma) using a Criterion blotter and Towbin buffer (Bio-Rad) at 10 V overnight in a cold room with stirring. Protein transfer was verified using Ponceau S staining (MilliporeSigma). Membranes were destained and blocked in 3% BSA/TBS-T (TBS containing 0.1% Tween 20; Thermo) for 45 min at RT, with rocking. Membranes were cut into strips and probed overnight (4°C, rocking) using mouse monoclonal primary antibodies diluted in 1% BSA/TBS-T. Membranes were washed 3 times with TBS-T and then incubated with anti-mouse IgG horseradish peroxidase(HRP)-linked secondary antibodies (Cell Signaling Technology) diluted 1:3,000–1:10,000 in 5% non-fat milk/TBS-T for 2 h at RT. Membranes were washed 3 times, then incubated in SuperSignal West Femto Maximum Sensitivity Substrate (Thermo) for 5 min and imaged using a ChemiDoc MP Imaging System (Bio-Rad).

Mouse monoclonal primary antibodies included: anti-CD63 (clone MX-49.129.5, 2 μg/mL), anti-tsg 101 (clone C-2, 2 μg/mL), anti-ApoA-I (clone 069–01, 1 μg/mL), anti-Histone cluster 1 H3D (clone 6H8, 1 μg/mL), anti-cytochrome c1 (clone A-5, 2 μg/mL), anti-GM130 (clone B-10, 2 μg/mL), anti α-actinin (clone H-2, 2 μg/mL), anti-eIF2C (clone B-3, 2 μg/mL), and anti-hnRNP A2/B1 (clone b-7, 2 μg/mL) from Santa Cruz Biotechnology (Dallas, TX); and anti-CD81 (clone 1.3.3.22, 2 μg/mL), anti-Alix (clone 3A9, 2 μg/mL) from Invitrogen.

### Lipidomic Mass Spectrometry

The MRM-profiling methodology was used as previously described (15, 16). Experiments were performed using an Agilent 6410 QQQ mass spectrometer (Agilent Technologies) with micro‐autosampler (G1377A). Lipid extraction was performed using the Bligh & Dyer protocol (17). Briefly, 200 μL of buffer containing the EV protein was combined with 450 μL and 250 μL of methanol and chloroform, respectively. After RT incubation for 15 min, 250 μL of ultrapure water and chloroform were added and samples were centrifuged to amplify the separation of the lipid, metabolite, and protein phases based on differences in polarity. The lipid (bottom) layer was extracted and dried under a stream of nitrogen and stored at -80°C until MS analysis.

The dried samples were then resuspended in appropriate volumes of acetonitrile (ACN)/methanol/ammonium acetate 300 mM, v/v/v, 6.65:3:0.35 (injection solvent). 8 μL of diluted EV lipid extract was injected into the electrospray ionization (ESI) source of the MS. The capillary pump connected to the autosampler operated at a flow rate of 10 μL/min and a pressure of 100 bar. Capillary voltage on the instrument was 3.5‐5 kV and the gas flow was 5.1 L/min at 300°C.

MRM-profiling is a two-phase process containing both discovery and screening phases. The representative sample pool used in the discovery phase consisted of 14 different EV samples from rat cell lines. For this phase, using methods previously reported by de Lima *et al*., we applied a list of 1,419 MRMs from 10 lipid classes: phosphatidylcholine (PC)/sphingomyelin (SM), phosphatidylethanolamine (PE), phosphatidylinositol (PI), phosphatidylglycerol (PG), phosphatidylserine (PS), ceramide, cholesteryl ester (CE), acyl-carnitine, free fatty acid (FFA), and triacylglycerol (TAG) (15). The monitoring of these classes was based on precursor ions of lipids listed in the Lipid Maps Database (http://www.lipidmaps.org/) and product ions common to each given lipid class.

Raw MS data, MRM transitions and intensities, were processed using in-house scripts in order to generate a list of MRM transitions and their respective ion intensities. Comparison of the absolute ion intensities for the EVs to a blank sample (injection solvent) was then assessed and MRMs with an ion intensity at least 30% higher than the blank were selected. The top 200 MRMs were selected for the screening phase and monitored over a period of 2 min per sample. The screening method included MRMs from five lipid classes (PC and SM, Cholesteryl esters, ceramides, PE) and a single metabolite (acyl-carnitine) class.

### *In vivo* Kinetic Experiments

Male Sprague–Dawley rats were implanted with jugular vein catheters (12) (Access Technologies, Skokie, IL) and provided a 2 d recovery period. Animals were weighed prior to dose administration and then again at euthanasia. A negative control blood sample was collected immediately prior to dosing, and a dose aliquot was reserved (frozen at -80°C) for later analysis. Catheter access was achieved by placing conscious animals in a rodent restrainer and using 1 mL tuberculin slip-tip syringes (BD) with attached blunt 22 ga dispensing needles (Jensen Global, Santa Barbara, CA). Blood samples were collected from each animal at 2, 7.5, 15, 30, 60, 120, 240, 480, 960, and 1440 min after dosing using syringes pre-loaded with 20 µL 4% sodium citrate. Each collection involved the following steps: discarding of lock solution and 0.1 mL blood, collection of 0.2 mL blood, pulsatile flushing of the catheter with 0.25 mL saline, and locking of the catheter with 0.1 mL 4% sodium citrate. Blood plasma was separated from the blood (2,000 × g, 20 min, 4°C) and then clarified (10,000 × g, 10 min, 4°C) by centrifugation. Two 50 μL aliquots were transferred to 1.5 mL microcentrifuge tubes and stored at -80°C.

The target dose amount was determined using EVs from clone 9 cells expressing XMc39 tracer. EVs were prepared in bulk and quantified by protein (see previous). RNA extracted from 100 μg EVs (2 μg/μL) was diluted 1:100 in water and analyzed by ddPCR. Using the estimated average total blood volume of 300–450 g male Sprague Dawley rats (18), we determined that 1,000 μg EVs at a concentration of 2 μg/μL would result in an initial plasma concentration (C_0_) near the upper limit of ddPCR detection. A preliminary *in vivo* time course was performed to validate the calculated dose amount, and to establish experimental duration.

High and low analytical standards were produced to capture the batch variability of RNA extraction and analysis, as follows. Citrated blood from two exsanguinated naïve animals was pooled. Labeled Clone 9 sEVs were added to a portion of naïve blood and mixed by inversion. An equivalent amount of PBS was added to the remaining naïve blood. Crude plasma was separated from the blood by centrifugation (2,000 × g, 20 min, 4°C) and then clarified (10,000 × g, 10 min, 4°C). Labeled plasma was serially diluted by unlabeled plasma and analyzed by ddPCR to identify an appropriate high standard concentration. The low standard was prepared by diluting the high standard 30-fold with unlabeled plasma. After final confirmation by ddPCR, 50 μL aliquots of each standard were prepared and stored at -80 C.

### Tracer miRNA Time Course Stability Assay

Blood for the *in vitro* experiment was collected from a euthanized male Sprague Dawley rat by cardiac puncture. Briefly, the animal was euthanized by isoflurane inhalation (5% induction, 5% maintenance) and a laparotomy was performed, followed by a bilateral anterolateral thoracotomy. One 20 mL syringe (cat. no. 309661, BD) pre-filled with 1 mL 4% sodium citrate (Fenwal) was used to obtain 10 mL blood from the exposed heart. The citrated blood was mixed by gentle inversion and 8 mL was transferred to a 15 mL LoBind conical tube (Eppendorf), then continuously warmed in a 37°C water bath. *In vitro* and *in vivo* time course experiments were performed in parallel, beginning with the administration of EVs. After dosing the conscious rat, 150 μL (300 μg) of the same EV dose preparation was spiked into the warmed anticoagulated blood and mixed by gentle inversion. Subsequent to each *in vivo* blood collection, an *in vitro* sample was collected from the anticoagulated blood (which was gently inverted to prevent red blood cell settling). *In vitro* blood samples were collected up to 240 min, and handled identically to the *in vivo* blood samples.

### Preparation of Standard Curve

Standard curves were independently performed using serial dilutions of the high standard. Since the high standard was designed to achieve a maximum copy concentration in an intermediate-high range (~ 25,000 copies/20 μL), a fivefold concentration was prepared by performing Qiazol phase separation for each of 5 aliquots and binding the RNA precipitates to the same silica membrane column prior to elution. From this 5X high standard, twofold dilutions were prepared using miRNA from naïve rat plasma as the diluent. Concentrations were obtained by ddPCR.

### Data Normalization

To account for technical variability, high and low standards were included with every set of samples analyzed by ddPCR. We normalized the data using our standard curve as follows. Standard curve copy numbers were plotted against their concentration factor; the concentration factors ranged from ~ 0.001 to 5. A linear regression of the standard curves was performed with Excel 2019 (Microsoft Corporation, Redmond, WA), and reference standard copy numbers were calculated for concentration factors of 1, corresponding to the 1X high standard, and $\text{0.0}\stackrel{\mathrm{-}}{3}$ corresponding to the low standard which is a 30-fold dilution of the high standard. For every set of samples, the internal high and low standards were used to normalize observed copy numbers to the reference standards. Copy number concentrations were then converted to EV protein concentrations and normalized against the dose aliquot for each set of samples, taking all dilution factors into account (Supplementary Fig. 5). Normalizing copy numbers to EV protein concentrations effectively accounts for differences between cell lines and potential variability between EV preparations.

### EV Pharmacokinetic Modeling

Modeling EV disposition following IV administration was performed using a population pharmacokinetic approach. Phoenix 64 build 8.1.0.3530 (Certara, Princeton, NJ) was used to support non-linear mixed effects analysis with first order conditional estimation—extended least squares (FOCE ELS) to estimate population-level parameters with associated inter-animal variability on those parameters. Initial parameter estimates were made using the “initial estimates” function in Phoenix to manually create the best fit lines to the observed data. Subsequently, each sequence of parameter estimation was limited to a maximum of 1,000 iterations. Observed concentrations were fit to the exponential form of equations describing two-compartment and three-compartment model structures (Fig. 4b). Equations were parameterized according to clearance between compartments and the compartment volumes. Inter-individual (IIV) random effects for the various structural parameters were included as a diagonal matrix initially. These random effects are reported as percent variance from a log-normal distribution of individual subject parameter estimates, the basis of which is the exponential relationship, P_i_ = P_tv_ x exp(η_i_), where P_i_ is the parameter estimate for the ith individual, P_tv_ is the population typical value, and η_i_ (eta) is the deviation from the population value for the ith subject. Correlation of IIVs among parameters was evaluated graphically to support the need to estimate covariance of random effects between parameters. A multiplicative (proportional) residual error model was applied using the relationship, Cobs = C * (1 + CEps)), where Cobs is the observed concentration, C the model predicted concentration and CEps the difference between Cobs and C. Covariates were multiplied to population-parameter estimates (thetas) exponentially as theta *e^covariate^. Evaluation of the final 3 compartment model with cell line covariates consisted of a prediction-corrected visual predictive check (pcVPC) of 1,000 simulations based on the final parameter estimates. Bootstrap analysis was used to evaluate parameter stability. For the pcVPC, a log-additive residual error model was used in place of a multiplicative error model. The log-additive model is the same as a multiplicative model, except that it prevents simulations resulting in negative EV concentrations, as negative concentrations are not possible. Simulated concentrations from the pcVPC were stratified by cell line, and the concentrations binned by k-means (the mean of the times). Median and associated 5% and 95% confidence limits of the observed EV concentrations were superimposed with their corresponding median predicted values and associated 5–95% intervals of these median predictions. The bootstrap analysis consisted of 1,000 samples with replacement from the original set of animals (each sample containing the same number of animals as the original study).

### Statistics

Sample sizes for this study were determined using data from Morshita et. al (4). A sample size of 10 rats would have 80% power to detect a 30% change in exosome clearance using an unpaired *t*-test and 5% type 1 error rate. This is estimated based on the calculation of EV clearance to be 0.52 ml/min, and a conservative estimate of 25% variability (given the limited data available). EV clearance was calculated using the following equations: 100% ID = 37 kBq; 37 kBq/100% ID × 3.2 (%ID x hr / mL) = 1.184 kBq x hr / mL = AUC; CL = d / AUC; CL = 37 kBq / 1.184 kBq x hr /mL; CL = 31.25 mL / hr; thus CL = 0.52 mL / min. CL = clearance, d = dose, hr = hour, AUC = area under the concentration–time curve; kBq = kilo Becquerel. We estimated this sample size and sampling frequency per animal was adequate to support non-linear mixed effects analysis.

Elimination half-life (T ½), compartment distribution half-life, and AUC were determined from the Phoenix post-hoc data for the final model. Elimination T ½ for each sample ln 2/(Cl/(V + V2 + V3)), compt 2 distrib T ½ = ln2 / (Cl2/V2). Compt 3 distrib T ½ = ln2 /(Cl3/V3). JMP Pro 14 was used for statistical analysis. Given a sample size of 10 and without the assumption of normal distribution or equal variance, Wilcoxon and Kruskal–Wallis rank-sum tests were applied as a conservative non-parametric approach to determining significant differences between cell lines (P < 0.05). If significance was met by the Wilcoxon/Kruskal–Wallis test, then the Steel–Dwass method was applied to evaluate for significant differences between cell lines. Steel–Dwass makes non-parametric comparisons for all pairs and takes into account multiple comparisons similar to Tukey’s Method for parametric data.

Lipidomic analysis was performed with MetaboAnalyst 4.0 (www.metaboanalyst.ca) using the following options. Sample ion counts were normalized by sum and auto-scaled. One-way ANOVA and Fisher’s LSD post-hoc analysis were performed to select top scoring lipids (unadjusted *P* < 0.05) for PCA and heat map.

## Results

### Preparation of Labeled Extracellular Vesicles

**Fig. 2:**
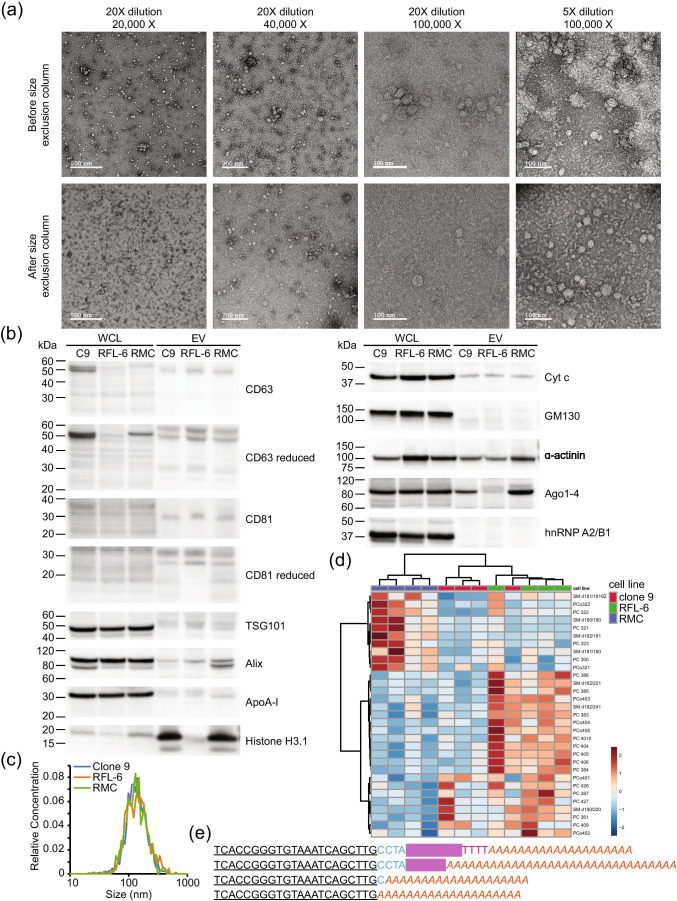
**Characterization of EVs.** (**a**) Transmission electron micrographs of EVs before and after purification by size exclusion centrifugation. The first three columns represent different imaging magnifications (20,000X, 40,000X, and 100,000X) with a sample dilution of 20X; the fourth column represents 100,000X magnification with a sample dilution of 5X. The top row represents unpurified samples, and the bottom row represents purified samples. (**b**) Western blots for EV and non-EV markers in whole cell lysates (WCL) and EV preparations (Clone 9, RMC, RFL-6). Molecular weight markers are designated by lines on the left of each blot. (**c**) Average size distributions of EVs from cultured clone 9 hepatocytes, RFL-6 lung fibroblasts, and RMC mesangial kidney cells. Average distribution was produced by taking average bin counts of 3 replicates per cell line. (**d**) Heat map representing the top 31 EV lipids, clustered by cell type (n = 4 for each cell line). Grouping was performed by unsupervised hierarchical cluster analysis (Euclidean distance, Ward linkage) of ion counts normalized to sum and auto-scaled. (**e**) EV-associated tracer miRNA products with variable length 3’ sequences. An expression vector-specific sequence (blue) separates the mature cel-miR-39-3p sequence (bolded and underlined) from the exosome localization signal (redacted in magenta). The first sequence includes a partial poly-T transcription termination sequence (red), which is encoded in the expression vector. Long poly-A tails (orange italics) are added during miRNA cDNA synthesis.

In order to discriminate exogenously administered EVs from endogenous background in rats, we incorporated a tracer miRNA sequence that did not share homology with known rat miRNAs. The chosen tracer miRNA was expressed using a commercial lentivector which appends an exosome localization motif (19) to the resulting mature miRNA. For early optimization experiments, we used a proprietary non-targeting sequence (XMIR-NT). During development, we encountered constraints that required a known sequence. We selected *C. elegans* miR-39-3p (cel-miR-39) for cloning into the same lentivector (Supplementary Fig. 1), designated “XMc39”. Because of its non-homology with many species, cel-miR-39 is commonly used as a quality control spike-in for miRNA PCR experiments involving biofluids from humans, rats, and other mammals (14, 20, 21). The validated XMc39 plasmid was transfected into 3 established rat-derived cell lines (clone 9 liver hepatocyte, RFL-6 lung fibroblast, and RMC kidney mesangial cells) which produced EVs labeled with tracer miRNA. EVs were isolated from enriched media using a commercial chemical isolation reagent (Fig. 1). Compared to ultracentrifugation, chemical reagents allow for substantially greater yield when retrieving EVs from biofluids and cell culture supernatants with the trade-off of lower purity (22). Co-precipitation of medium to large vesicles (3) was minimized by including an additional 10,000 × g centrifugation step (23, 24) prior to addition of reagent. Residual chemical reagent was removed by careful washing of the pellet, resuspension, and filtration through low molecular weight size exclusion columns (Fig. 2a). Nanoparticle tracking analysis and transmission electron microscopy confirm that 86.5% ± 1.5% (mean ± S.E.) of all particles are in the 45–195 nm size range (Figs. 2a,c; Supplementary Fig. 2) of exosome-enriched small EV (sEV) preparations, and deprived of aggregates (Fig. 2a). Western blot analysis (Fig. 2b) demonstrates the presence and absence of sEV-associated proteins and relevant co-precipitated non-sEV contaminants (3, 19) in comparison to whole cell lysates, based on MISEV 2018 recommendations (3). Tetraspanins CD63 and CD81 (category 1a (3)) were represented in all EVs, consistent with other reports of reagent-based EV isolation methods (23, 25–29). Cytosolic membrane-binding proteins Alix and tsg 101 (category 2a (3)) were detectable in all EV samples. Apolipoprotein and mitochondrial markers ApoA-I (category 3a (3)) and cytochrome c (category 4b (3)) were present in EV samples, though less abundantly relative to whole cells. The secretory pathway (Golgi) marker GM130 (category 4c (3)) was absent in EV samples. Histone H3.1 (category 4a (3)) was particularly enriched in two EV samples. Histones may be associated with sEVs (30–32), with some evidence to the contrary (33). Cytoskeletal marker α-actinin (category 4d (3)) was present in all samples, indicating possible co-precipitation of autophagosomes. Two secreted non-vesicular miRNA-binding proteins (category 5 (3)) were assayed. Argonaute1-4 were detectable in two EV samples and non-sumoylated hnRNP A2/B1 (~ 35 kDa) (Supplementary Fig. 1) was barely detectable in one EV sample. Mass spectrometry confirmed that our EV preparations were enriched in sphingolipids and cholesterols (Supplementary Fig. 3, Supplementary Data 1), typical of exosomes (34), and indicated differences in lipid composition between EVs from each cell line (Fig. 2d).

**Fig. 3:**
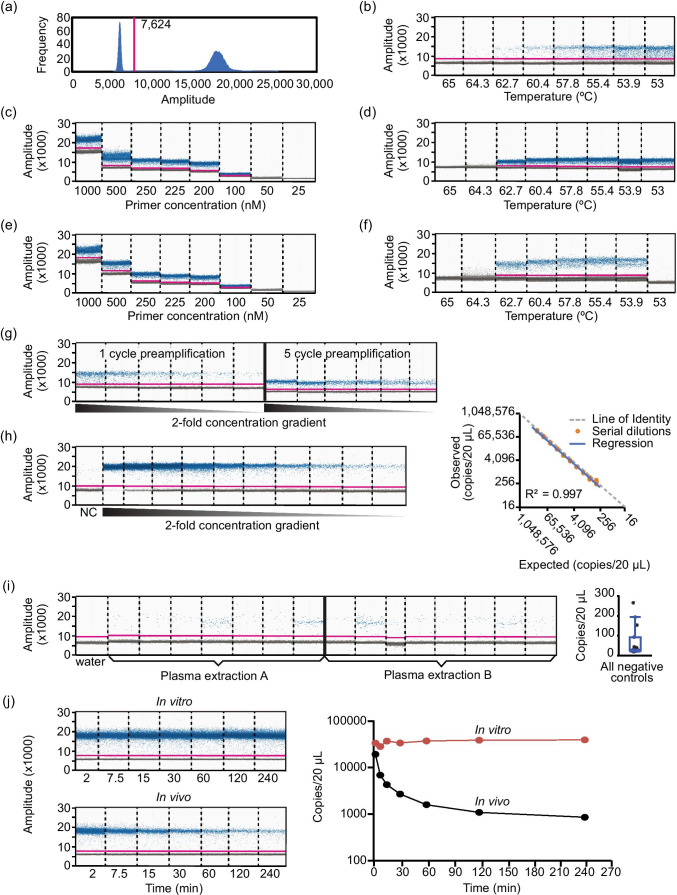
**ddPCR assay design and optimization.** (**a**) Representative histogram of EvaGreen fluorescence used to set a threshold (magenta line) between positive (left peak) and negative (right peak) droplets. (**b**) Amplitude scatterplot of initial annealing temperature (Ta) gradient (65 – 53°C) using XMIR-NT primer (100 nM) and fixed amount of XMIR-NT cDNA template. (**c**) Primer gradient (25—1000 nM); Ta = 60°C. (**d**) Ta gradient (65 – 53°C) using 250 nM primer; negative control (water) in the 65°C well. (**e**) Primer gradient; Ta = 58°C. (**f**) XMc39 (200 nM) Ta gradient (65 – 53°C). (**g**) Comparison between 1- and 5-cycle PCR pre-amplification. (**h**) Standard curve and evaluation of assay linearity. Concentrations from two-fold serial dilutions of XMc39 RNA (left) were used to plot expected vs. observed values (right). Individual observed concentrations (orange circles) and linear regression (blue line) closely aligned with the line of identity (dashed gray line). (**i**) Technical replication of negative control plasma samples; ddPCR (left) using n = 7 and n = 8 technical replicates from two separate RNA extractions, and boxplot (right) of concentrations. (**j**) Evaluation of EV-encapsulated tracer miRNA stability over time in anticoagulated whole blood at 37°C (left, top) or intravenously administered to a Sprague Dawley rat (left, bottom). Semi-logarithmic concentration vs time (right) demonstrating stability of XMc39 tracer miRNA *in vitro* (orange line) and *in vivo* (black line) over 240 min (n = 1).

### Droplet Digital PCR Assay Development and Optimization

For pharmacokinetic analysis, we wanted an assay with a dynamic range of five half-lives that could detect very low-abundance tracer miRNA during terminal phase kinetics. Droplet digital PCR (ddPCR) is more sensitive than quantitative PCR (35). The additional 3’ exosome localization sequence in our XMc39 tracer was incompatible with commercial TaqMan-based cel-miR-39-3p assays (Fig. 2e). We therefore designed an assay for use with the EvaGreen intercalating fluorophore. Conditions for ddPCR were optimized as follows.

Using cDNA synthesized from a known quantity of purified RNA template, we optimized PCR primer concentration and annealing temperature (T_a_). Early optimization used XMIR-NT with corresponding forward primer, supplied by the vendor. Starting with a conservative primer concentration (100 nM), we determined the T_a_ of 60°C was the highest temperature to give a positive droplet band (Fig. 3b). With a T_a_ of 60°C, we tested a primer concentration gradient (Fig. 3c). The optimal primer concentration (200–250 nM) was based on positive and negative band discrimination, percentage of positive droplets, and tight clustering of individual positive and negative bands. Next, we chose a primer concentration (250 nM), repeated a T_a_ gradient (Fig. 3d), and established T_a_ of 58°C as optimal. Finally, using a 58°C Ta, we repeated the primer concentration gradient (Fig. 3e) with an optimal primer concentration of 200–225 nM. We chose 200 nM to minimize nonselective EvaGreen fluorescence. The proprietary XMIR-NT sequence was replaced with cel-miR-39-3p (XMc39). Based on similarity in sequence length with the XMIR-NT forward primer, we used the same primer concentration of 200 nM to perform a final temperature gradient for XMc39 (Fig. 3f) and determine 56°C to be optimal.

Commercial cDNA synthesis kits typically include single-stranded oligo(dT) adapters in the 2–5 μM range. These kits are not optimized for use with ddPCR, and the high amount of oligo(dT) carryover creates non-selective EvaGreen fluorescence in droplets. We preamplified the cDNA to dilute oligo(dT) without sacrificing sensitivity (Fig. 3g). This improved our sensitivity and minimized the droplets between negative and positive negative bands (also known as rain) (Fig. 3g).

Assay linearity was determined by two-fold serial dilutions of a synthetic RNA oligonucleotide of the XMc39 sequence mixed with miRNA extracted from naïve rat plasma (to simulate biological background noise). Expected ddPCR copy number values from known amounts of XMc39 oligonucleotide were compared to observed values. As shown in Fig. 3h (n = 3), the relationship between expected and observed copy numbers was highly linear (r^2^ = 0.997) and nearly identical (compared to the line of identity).

Negative controls consisting of naïve plasma produced low, variable numbers of false positive droplets. To explore if this was a suitable determine the lower limit of quantification, we prepared miRNA from two negative control plasma samples and analyzed replicate aliquots of each. These samples produced a random signal ranging from 20 to 266 copies with a CV of 110% (Fig. 3i), as opposed to water controls which yielded no more than 1 positive droplet. Interestingly, we found that very low (undetectable) amounts of positive control RNA template added to negative control samples reduced the number and variability of false positive droplets (Supplementary Fig. 4). We decided to use negative controls as a measure of quality control rather than a hard threshold for data exclusion. Sample sets with negative controls greater than 200 were reanalyzed using RNA as the starting material. For this analysis, we did not define a lower limit of quantification and allowed the model to use all the data.

**Fig. 4:**
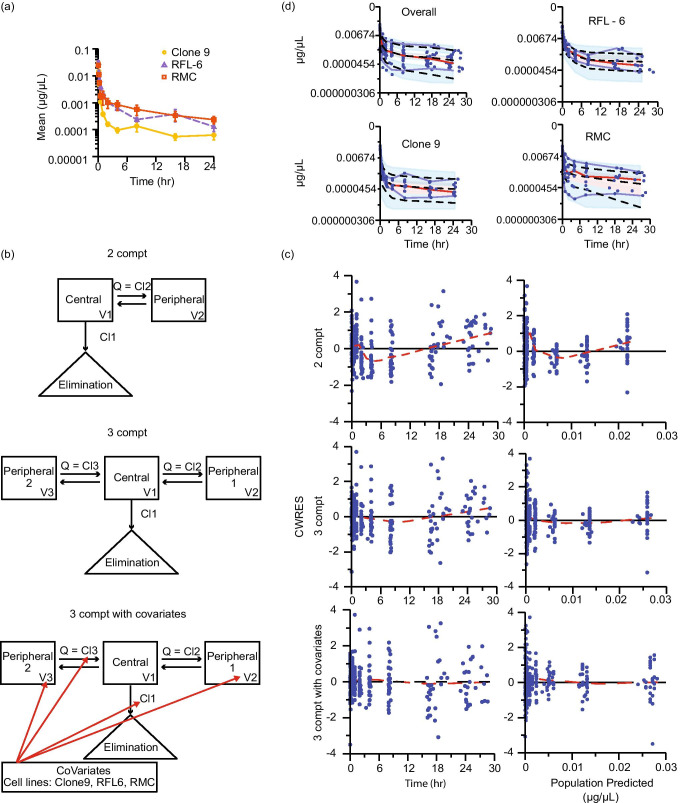
**EV kinetic modeling.** Final kinetic models for EVs administered to conscious Sprague Dawley rats. Each EV preparation had 9—10 animals (clone 9, n = 10; RFL-6, n = 9; RMC, n = 9). (**a**) Mean normalized EV concentrations (± SE) over time after single intravenous bolus dose. Semi-logarithmic plot illustrating *in vivo* time course data for EVs isolated from clone 9 (yellow circles), RFL-6 (purple triangles), and RMC (orange squares) cell lines. (**b**) Schematic representations of models: two-compartment (2 compt), three-compartment (3 compt), and three-compartment with covariates applied (3 compt with covariate). V = volume, Q = equal flow between two compartments (Phoenix software designates Q as numbered Cl parameters, e.g. Cl2 and Cl3). Red arrows indicate parameters to which the covariate was applied. (**c**) Goodness-of-fit plots for conditional population weighted residuals (CWRES) vs. time and CWRES vs. population predicted concentrations. Model schematics are placed to the left of the respective plots. Blue circles indicate CWRES, black line indicates the zero residual reference line, dashed red line indicated the LOESS regression. See Supplementary Fig. 6 for additional Goodness-of-fit plots. (**d**) Observation-based simulated posterior predictive evaluation with prediction-corrected visual predictive check (pcVPC) as semi-exponential plot of concentration vs. time. From top to bottom, the plots include data from all cell lines, Clone 9, RFL-6, and RMC. Observed measures include individual observations (blue circles), median (dashed red line), and 5th and 95th percentiles (blue lines). Predicted measures include median, 5th, and 95th percentiles (dashed black lines). Shaded ribbons indicate predicted 90% confidence intervals around each quantile.

### Stability of EVs *In Vivo*

Since blood plasma is rich in RNases that degrade unprotected circulating RNAs (data not shown) (36–38), we determined that our EV preparation protected the tracer miRNA from nonspecific RNase degradation. For the stability assay, EVs labeled with tracer miRNA were intravenously administered to a live rat (*in vivo*); in parallel, an amount (proportional to rat blood volume) of the same EVs was spiked into a tube of anticoagulated whole blood (*in vitro*). The whole blood was incubated at 37°C (to mimic body temperature). During the course of the experiment, *in vitro* blood samples were drawn from the tube immediately after *in vivo* blood samples at pre-specified time intervals (Fig. 3j). Tracer miRNA was stable *in vitro* for the 4 h time course,while rapidly eliminated *in vivo* over the same 4 h. We concluded that the detectable tracer miRNA in the EV preparations was protected from RNAse degradation in the blood.

### *In Vivo* Kinetics of Intravenously Administered Extracellular Vesicles

This optimized method was applied to test our hypothesis that EVs from different cultured cell lines of origin exhibit different kinetics. Three Sprague Dawley-derived cell lines were selected for this study: clone 9 liver hepatocytes, RFL-6 lung fibroblast, and RMC kidney mesangial cells. Liver, lungs, and kidneys have been identified as major organs of exosome clearance (39–43). EV preparations from each cell line were administered to 10 animals; thus, 30 animals were used in total. Blood samples were collected from each and analyzed in batches (Supplementary Fig. 5, Supplementary Data 2). Two animals were excluded from analysis. One animal from the RFL-6 group was removed for concern of cross-sample contamination, and one animal from the RMC group for failing quality control according to the pre-defined negative control threshold.

The unmodeled data, consisting of normalized observed concentrations plotted against the ideal collection time, showed differences in EV kinetics between cell lines as visually represented on a semi-log plot (Fig. 4a) and appeared to be multi-exponential, likely tri-exponential. For compartmental analysis, we used first order conditional estimation—extended least squares (FOCE ELS) to estimate pharmacokinetic parameters. A one-compartment model would not execute in the modeling software. As reported in Table I, a three-compartment model with one elimination from the central compartment (“3 compt model”) results in a much lower Akaike information criterion (AIC) value than a two-compartment model with one elimination from the central compartment (“2 compt model”) (Table I). Models with elimination from the central compartment are the simplest models (44) (Fig. 4b), and likely exhibit the lowest AIC values because we only analyzed tracer miRNA concentrations in blood sampled from the central circulation.

**Table I:** Model comparisons

Model	Akaike Information Criterion (AIC)
1 compartment and 1 to 3 eliminations	Unable to estimate parameters
2 compartment 1 elimination (central compt)	-3268.419
2 compartment 2 elimination	-3271.057
2 compartment 1 elimination (peripheral compt)	-1864.035
2 compartment 3 elimination (2 central, 1 peripheral compt)	-3260.423
3 compartment 1 elimination (central)	-3324.393
3 compartment 1 elimination (central)—cell line covariate applied to all parameters	-3364.679
3 compartment 1 elimination (central) – cell line applied to V2, V3, Cl, Cl3	**-3367.219**
3 compartment with 2 elimination (both peripheral)	-3313.1419
3 compartment with unequal distribution rates between compt and 1 elimination (central)	-3328.015 ^a^
3 compartment – two unequal distribution rates between compt—1 elimination (central)	
Cell line covariate applied to all parameters	-3321.4789 ^a^
3 compartment 3 elimination	-3312.0896

a – No precision estimates for parameters

Covariates of cell line, weight, and batch were incorporated into the three-compartment model, and only the cell line covariate resulted in a meaningful decrease in the AIC value and change in eta-covariate comparisons. Using a shotgun approach of applying the cell line covariate to each parameter, we found that applying the covariate to Volume 2 (V2), Volume 3 (V3), Clearance (Cl), and Clearance 3 (Cl3) (Fig. 4b) resulted in the lowest AIC value (Table I). Code for execution of the model can be found in Supplementary Note.

### Population Model Evaluation

We compared goodness of fit scatterplots between the two- and three-compartment models (Fig. 4c; Supplementary Fig. 6). Model fitness is improved as the LOESS regression line approaches the ideal weighted residual line of zero. In evaluating the conditional population weighted residual (CWRES) *versus* time and *versus* the population predicted concentrations, the three-compartment model improved the model fit of the data (Fig. 4c). In evaluating observed concentrations *versus* individual predicted concentrations and population predicted concentrations, the LOESS regression line approached the line of unity indicating that the three-compartment model again outperformed the two-compartment model (Supplementary Fig. 6). The addition of the covariates to the three-compartment model further improved upon the base model (Fig. 4c, Supplementary Fig. 6) with individual model fits in Supplementary Fig. 7.

We performed an observation-based simulated posterior predictive evaluation with prediction-corrected visual predictive check (pcVPC, Fig. 4d) using a log-additive error model to prevent simulating negative concentrations. The simulated three-compartment model with covariates contains the observed data within the shaded confidence interval, which suggesting a good model description.

### Model Outcome and Performance

Notably, the volume of distribution in the central compartment (28 mL) is similar to the mean calculated total blood volume of a male Sprague Dawley rat (18) with an average weight of 372 ± 6 g, or 26 ± 0.4 mL (mean ± S.E.). As shown in Table II, the half-life of elimination ranged from 12 to 215 h across the 3 cell lines and was significantly different between them. The volume of distribution between the central compartment and first peripheral compartment was significantly different between the clone 9 and RFL-6 cell lines. The volume of distribution between the central compartment and second peripheral compartment was significantly different between all cell lines. The area under the concentration–time curve (AUC) was significantly different between RMC and clone 9, and between RMC and RFL-6.

**Table II:** Population Pharmacokinetic Parameter Estimates Describing Plasma Exosome Exposure Following Intravenous Administration

	3 compartment model estimate					Boot strap				T_1/2_ elim. (hr)	n	Mean/Median		90%CI	Comparisons		p value
	Parameter	Estimate		95% CI		Mean		95% CI		clone 9	10	24	22	(3—52)	RFL-6	clone 9	**0.011**
Clone 9	V1	28	mL	(24—31)		28	mL	(24—31)		RFL-6	9	149	128	(93—205)	RMC	clone 9	**0.042**
Clone 9	V2	3,057	mL	(1,351—4,762)		4,057	mL	(1,053—8,200)		RMC	9	9	9	(6—11)	RMC	RFL-6	**0.010**
Clone 9	V3	16	mL	(4—28)		18	mL	(9—34)									
Clone 9	Cl1	93	ml/hr	(56—131)		86	ml/hr	(43—135)		Compt 2 T_1/2_ (hr)							
Clone 9	Cl2	111	ml/hr	(80—143)		118	ml/hr	(76—156)		clone 9	10	20	17	(13—27)	RFL-6	clone 9	0.72
Clone 9	Cl3	21	ml/hr	(15—27)		22	ml/hr	(15—31)		RFL-6	9	37	38	(17—57)	RMC	clone 9	**0.017**
RFL 6	V2	2,982	mL	(1,315—4,657)		3,923	mL	(902—8,684)		RMC	9	5	4	(2—8)	RMC	RFL-6	**0.013**
RFL 6	V3	90	mL	(10—337)		95	mL	(23—349)									
RFL 6	Cl1	19	ml/hr	(5—68)		0	ml/hr	(0—75)		Compt 3 T_1/2_ (min)							
RFL 6	Cl3	124	ml/hr	(60—236)		124	ml/hr	(49—294)		clone 9	10	31	31	(31—32)	RMC	RFL-6	**0.0012**
RMC	V2	752	mL	(166—2,353)		881	mL	(83—6,012)		RFL-6	9	30	30	(30—30)	RFL-6	clone 9	**0.0008**
RMC	V3	17	mL	(4—31)		21	mL	(6—123)		RMC	9	11	11	(11—11)	RMC	clone 9	**0.0008**
RMC	Cl1	66	ml/hr	(35—106)		66	ml/hr	(13—283)									
RMC	Cl3	63	ml/hr	(20—186)		77	ml/hr	(15—668)		AUC (ug*hr/mL)							
	Stdev0 =	0.45				Stdev0 =	0.44			clone 9	10	10	9	(8—13)	RFL-6	clone 9	**0.0081**
										RFL-6	9	60	60	(40—80)	RMC	RFL-6	0.51
										RMC	9	22	17	(11—32)	RMC	clone 9	**0.028**

For each EV source, results from the optimized 3-compartment model are presented as the average value with associated 5% and 95% limits of the 90% confidence interval of the estimate. Associated parameter stability from a bootstrap analysis (1,000 simulations) are also presented. Statistical analysis results of between-source comparisons of half-life and total exposure (AUC) are also summarized. V refers to volume of distribution of a compartment; Cl refers to elimination clearance from compartment 1 (Cl1), or between compartment 1 and 2 (Cl2) or 1 and 3 (Cl3) distributional clearance. N (n) refers to the number of animals

A bootstrap analysis using 1,000 simulations was performed to evaluate the likelihood of achieving similar results if the experiment was replicated. Overall, the bootstrapped results mirrored the actual experiments. One exception is that the clearance of elimination from the central compartment (Cl) was similar for all 3 cell lines (Table II, Supplementary Fig. 8). This suggests that cell line differences in EV kinetics are due to differences in EV distribution to the peripheral compartments.

## Discussion

EVs continue to attract broad interest as both targeted therapeutics and dynamic biomarkers in the systemic circulation, yet many modalities for the study of *in vivo* EV kinetics are focused on modifications of membrane composition that provide a partial picture of how composition affects kinetics. Here, we provide a method for modeling the *in vivo* kinetics of EVs derived from cultured cells. We integrate several techniques in this approach. First, an expression vector is used to encode a non-homologous tracer miRNA that is packaged into small exosomes. Second, EVs labeled with tracer miRNA are harvested from cell culture media and studied *in vivo* through injection into rats. Third, droplet digital PCR is used for the large dynamic range of detection of tracer miRNA from low-volume blood samples. This approach is ideal for EV kinetic modeling by 1) not introducing steric hindrances on the sEV membranes; 2) allowing for all time course samples to be drawn from one animal to reduce intraindividual variability; and 3) providing a wide dynamic range of detection to discern differences EV kinetic profiles.

We tested the hypothesis that EVs from different cell lines exhibit different kinetics *in vivo*. These studies quantitatively described significant differences in kinetic parameters between EVs from three different cell lines. In general, EVs have multiple routes of elimination (e.g. tissue sequestration, intracellular degradation, and excretion). Since we sampled blood from the central blood compartment, we did not have enough data to model elimination from peripheral compartments. Our three-compartment model supports the idea that EVs circulate in the vasculature and then move between two compartments; a shallow (small volume) and deep (large volume) peripheral compartments that may represent intervascular distribution to white blood cells and tissue distribution respectively.

A three-compartment model best described the observed kinetics of EVs derived from all three cell lines used in this study. While there are reproducible differences in sEV kinetics when comparing the three cell lines across multiple passages, some caution should be used when interpreting the reasons why. The effect of cell type on the model may reflect covariables other than cell type. This includes differences in the cell line-specific culture media used in our study and the percentage of FBS used. Our results indicate cell-line related differences when those cell lines are cultured under ATCC recommended conditions. The strategy we’ve presented may also be used to study the effects of various culture conditions on EV kinetics from a given cell line.

Isolating EVs using PEG precipitation-based methods may yield samples with lower purity compared to ultracentrifugation (25, 29, 45). Although our tracer miRNA includes a localization signal to selectively target EVs (19), other co-isolated miRNA-binding proteins may carry and protect tracer miRNA from RNAse degradation. Western blots demonstrated the presence and absence of two such proteins in our EV preparations, Argonaute (36, 46) and hnRNPA2/B1 (47), respectively. To our knowledge, there have been no reports or proposed mechanisms for non-vesicular miRNA-binding proteins to display altered elimination kinetics based on cell type. EVs in our study were isolated using the same procedure regardless of cell type, so any potential non-specific effects on EV kinetics due to PEG should remain constant. Thus, significant differences in the compartmental and non-compartmental EV kinetics between cell lines in our analysis are valid, though the amount of starting tracer in EV preparations may differ based on the relative purity of those samples.

We established the inability of negative control (naïve) plasma to accurately define a lower limit of quantification due to background noise. Our data suggest random off-target PCR amplification occurs when there is a lack of template and this effect is reduced when target template is present, even at very low concentrations.

The biological fate of cel-miR-39 packaged into cell culture-derived sEVs intravenously administered to conscious rats is not specifically known. Considerable effort has been made to evaluate the storage stability of EVs in biofluids and after isolation (48, 49), but very little is known about EV stability *in vivo*. There is some evidence to indicate that EVs stored in blood or plasma are somewhat more stable than isolated EVs at 4°C (48), suggesting the presence of structurally protective factors in the blood. Our own work is in agreement with other measurements of labeled EV kinetics within 4 h of administration to animals (4, 6, 9), and demonstrates the improved sensitivity of our method at later time points. Plasma is abundant with RNAse and circulating miRNA is rapidly degraded unless protected from degradation by extrinsic factors (50) such as bound proteins and encapsulation within vesicles (46). In blood samples stored at room temperature, endogenous miRNA was stable over 12 h (13). In the present study, isolated sEVs were added to fresh, anticoagulated blood and the packaged tracer miRNA was stable for at least 4 h at 37°C (Fig. 3j). Intrinsically, RNA stability is dependent on factors such as pH, temperature, and length (51); under physiological conditions, RNAse-protected miRNA would remain stable well beyond the duration of our study. Altogether, the evidence indicates physical stability of both sEV and its packaged miRNA throughout the studied time course.

Clinical implementation of EVs remains complicated by wide heterogeneity in membrane composition, contents, and cellular origin (7, 8). Recent breakthroughs have demonstrated the ability to predict and measure interindividual variability in drug metabolism using EVs derived from human blood samples (52, 53). In one case, analysis of hepatic EV contents was used to test tailored dosing regimens in silico (52), while in the other, EVs were used to directly measure the activity of drug metabolism enzymes (53). In terms of therapeutics, however, there is little information on how EV membrane components affect the pharmacokinetic parameters of custom-tailored EVs from cell culture bioreactors. In terms of biomarkers, it is difficult to assess the proper timing for maximal signal-to-noise without an understanding of when the relevant EVs will be at peak concentration in the blood. Our work was premised on the idea that endogenous circulating EVs exhibit steady-state kinetics, determined by rates of secretion into and clearance out of the blood. In order to quantitatively decipher the complexity of circulating EVs, we devised a method to determine the kinetic parameters of EVs from known origins. In this way, we can begin to systematically approach the identification of membrane components which affect EV kinetics in the blood. Limitations of our work include the following: 1) We have presented an *in vitro* to *in vivo* animal study, 2) our data are limited to sEVs derived from three cell lines, and 3) our method is currently difficult to adapt for use in humans due to cost and safety. Despite these limitations, our process fits nicely into preclinical animal studies where EV composition and kinetics can be evaluated to support the rational development of human EV therapeutics and biomarkers. Functional association of EV membrane components to kinetic parameters will allow other researchers to identify specific molecules that contribute to EV behavior in circulation.

## Conclusion

We studied *in vivo* clearance of EVs isolated from cultured cells using an internalized oligonucleotide tracer and modeled kinetic differences between EVs from different cell lines. We hope for this to be a tool in systematic approaches for studying differences in EV kinetics, such as when EVs are engineered with specific surface receptors/ligands for therapeutics. This approach has the potential to be combined with tissue distribution time course studies in physiologically-based systems biology approaches. By using conventional techniques and reagents, our method can be tailored to address a variety of scientific questions.

## Supplementary Information

Below is the link to the electronic supplementary material.

Supplementary file1 (XLSX 70 kb)

Supplementary file2 (XLSX 351 kb)

Supplementary file3 (PDF 1729 kb)

Supplementary file4 (PDF 54 kb)

## Data Availability

Data analyzed during this study are included in the published article and its supplementary information files. Raw data (mass spectrometry and electron micrographs) are available from the corresponding author upon reasonable request.

## References

[1] Raposo G, Stahl PD (2019). Extracellular vesicles: a new communication paradigm?. Nat Rev Mol Cell Biol.

[2] Rodrigues D, Rowland A (2019). From endogenous compounds as biomarkers to plasma-derived nanovesicles as liquid biopsy; has the golden age of translational pharmacokinetics-absorption, distribution, metabolism, excretion-drug-drug interaction science finally arrived?. Clin Pharmacol Ther.

[3] Thery C, Witwer KW, Aikawa E, Alcaraz MJ, Anderson JD, Andriantsitohaina R (2018). Minimal information for studies of extracellular vesicles 2018 (MISEV2018): a position statement of the International Society for Extracellular Vesicles and update of the MISEV2014 guidelines. J Extracell Vesicles.

[4] Morishita M, Takahashi Y, Nishikawa M, Sano K, Kato K, Yamashita T (2015). Quantitative analysis of tissue distribution of the B16BL6-derived exosomes using a streptavidin-lactadherin fusion protein and iodine-125-labeled biotin derivative after intravenous injection in mice. J Pharm Sci.

[5] Saunderson SC, Dunn AC, Crocker PR, McLellan AD (2014). CD169 mediates the capture of exosomes in spleen and lymph node. Blood.

[6] Takahashi Y, Nishikawa M, Takakura Y (2016). Analysis and control of in vivo kinetics of exosomes for the development of exosome-based DDS. Yakugaku Zasshi.

[7] Willms E, Cabanas C, Mager I, Wood MJA, Vader P (2018). Extracellular vesicle heterogeneity: subpopulations, isolation techniques, and diverse functions in cancer progression. Front Immunol.

[8] Willms E, Johansson HJ, Mager I, Lee Y, Blomberg KE, Sadik M (2016). Cells release subpopulations of exosomes with distinct molecular and biological properties. Sci Rep.

[9] Takahashi Y, Nishikawa M, Shinotsuka H, Matsui Y, Ohara S, Imai T (2013). Visualization and in vivo tracking of the exosomes of murine melanoma B16-BL6 cells in mice after intravenous injection. J Biotechnol.

[10] Institute for Laboratory Animal Research (2011). Guide for the care and use of laboratory animals.

[11] Office of Laboratory Animal Welfare. Public Health Service Policy on Humane Care and Use of Laboratory Animals. Bethesda (MD): National Institutes of Health; 2015.

[12] De Luca T, Szilagyi KL, Hargreaves KA, Collins KS, Benson EA (2018). Improving the patency of jugular vein catheters in sprague-dawley rats by using an antiseptic nitrocellulose coating. J Am Assoc Lab Anim Sci.

[13] Benson EA, Skaar TC (2013). Incubation of whole blood at room temperature does not alter the plasma concentrations of microRNA-16 and -223. Drug Metab Dispos.

[14] Benson EA, Skaar TC, Liu Y, Nephew KP, Matei D. Carboplatin with decitabine therapy, in recurrent platinum resistant ovarian cancer, alters circulating mirnas concentrations: a pilot study. PLoS One. 2015;10(10):e0141279. 10.1371/journal.pone.0141279 PMC4612782 26485143

[15] de Lima CB, Ferreira CR, Milazzotto MP, Sobreira TJP, Vireque AA, Cooks RG (2018). Comprehensive lipid profiling of early stage oocytes and embryos by MRM profiling. J Mass Spectrom.

[16] Dipali SS, Ferreira CR, Zhou LT, Pritchard MT, Duncan FE (2019). Histologic analysis and lipid profiling reveal reproductive age-associated changes in peri-ovarian adipose tissue. Reprod Biol Endocrinol.

[17] Bligh EG, Dyer WJ (1959). A rapid method of total lipid extraction and purification. Can J Biochem Physiol.

[18] Probst RJ, Lim JM, Bird DN, Pole GL, Sato AK, Claybaugh JR (2006). Gender differences in the blood volume of conscious Sprague-Dawley rats. J Am Assoc Lab Anim Sci.

[19] Villarroya-Beltri C, Gutierrez-Vazquez C, Sanchez-Cabo F, Perez-Hernandez D, Vazquez J, Martin-Cofreces N (2013). Sumoylated hnRNPA2B1 controls the sorting of miRNAs into exosomes through binding to specific motifs. Nat Commun.

[20] Brunet-Vega A, Pericay C, Quilez ME, Ramirez-Lazaro MJ, Calvet X, Lario S (2015). Variability in microRNA recovery from plasma: Comparison of five commercial kits. Anal Biochem.

[21] Poel D, Buffart TE, Oosterling-Jansen J, Verheul HM, Voortman J. Evaluation of several methodological challenges in circulating miRNA qPCR studies in patients with head and neck cancer. Exp Mol Med. 2018;50(3):e454. 10.1038/emm.2017.288 PMC5898892 29520111

[22] Patel GK, Khan MA, Zubair H, Srivastava SK, Khushman M, Singh S (2019). Comparative analysis of exosome isolation methods using culture supernatant for optimum yield, purity and downstream applications. Sci Rep.

[23] Rider MA, Hurwitz SN, Meckes DG (2016). ExtraPEG: a polyethylene glycol-based method for enrichment of extracellular vesicles. Sci Rep.

[24] Szatanek R, Baran J, Siedlar M, Baj-Krzyworzeka M (2015). Isolation of extracellular vesicles: Determining the correct approach (Review). Int J Mol Med.

[25] Antounians L, Tzanetakis A, Pellerito O, Catania VD, Sulistyo A, Montalva L (2019). The regenerative potential of amniotic fluid stem cell extracellular vesicles: lessons learned by comparing different isolation techniques. Sci Rep.

[26] Jeon H, Kang SK, Lee MS. Effects of different separation methods on the physical and functional properties of extracellular vesicles. PLoS One. 2020;15(7):e0235793. 10.1371/journal.pone.0235793 PMC7340315 32634162

[27] Karttunen J, Heiskanen M, Navarro-Ferrandis V, Das Gupta S, Lipponen A, Puhakka N (2019). Precipitation-based extracellular vesicle isolation from rat plasma co-precipitate vesicle-free microRNAs. J Extracell Vesicles.

[28] Royo F, Zuniga-Garcia P, Sanchez-Mosquera P, Egia A, Perez A, Loizaga A (2016). Different EV enrichment methods suitable for clinical settings yield different subpopulations of urinary extracellular vesicles from human samples. J Extracell Vesicles.

[29] Tang YT, Huang YY, Zheng L, Qin SH, Xu XP, An TX (2017). Comparison of isolation methods of exosomes and exosomal RNA from cell culture medium and serum. Int J Mol Med.

[30] Muhsin-Sharafaldine MR, Saunderson SC, Dunn AC, Faed JM, Kleffmann T, McLellan AD (2016). Procoagulant and immunogenic properties of melanoma exosomes, microvesicles and apoptotic vesicles. Oncotarget.

[31] Ochieng J, Nangami G, Sakwe A, Rana T, Ingram S, Goodwin JS (2018). Extracellular histones are the ligands for the uptake of exosomes and hydroxyapatite-nanoparticles by tumor cells via syndecan-4. FEBS Lett.

[32] Takahashi A, Okada R, Nagao K, Kawamata Y, Hanyu A, Yoshimoto S (2017). Exosomes maintain cellular homeostasis by excreting harmful DNA from cells. Nat Commun.

[33] Jeppesen DK, Fenix AM, Franklin JL, Higginbotham JN, Zhang Q, Zimmerman LJ, *et al*. Reassessment of Exosome Composition. Cell. 2019;177(2):428–45 e18. 10.1016/j.cell.2019.02.029 PMC6664447 30951670

[34] Kreimer S, Belov AM, Ghiran I, Murthy SK, Frank DA, Ivanov AR (2015). Mass-spectrometry-based molecular characterization of extracellular vesicles: lipidomics and proteomics. J Proteome Res.

[35] Taylor SC, Laperriere G, Germain H (2017). Droplet Digital PCR versus qPCR for gene expression analysis with low abundant targets: from variable nonsense to publication quality data. Sci Rep.

[36] Arroyo JD, Chevillet JR, Kroh EM, Ruf IK, Pritchard CC, Gibson DF (2011). Argonaute2 complexes carry a population of circulating microRNAs independent of vesicles in human plasma. Proc Natl Acad Sci U S A.

[37] Turchinovich A, Weiz L, Langheinz A, Burwinkel B (2011). Characterization of extracellular circulating microRNA. Nucleic Acids Res.

[38] Vickers KC, Palmisano BT, Shoucri BM, Shamburek RD, Remaley AT (2011). MicroRNAs are transported in plasma and delivered to recipient cells by high-density lipoproteins. Nat Cell Biol.

[39] Aliotta JM, Pereira M, Sears EH, Dooner MS, Wen S, Goldberg LR (2015). Lung-derived exosome uptake into and epigenetic modulation of marrow progenitor/stem and differentiated cells. J Extracell Vesicles.

[40] Conde-Vancells J, Rodriguez-Suarez E, Embade N, Gil D, Matthiesen R, Valle M (2008). Characterization and comprehensive proteome profiling of exosomes secreted by hepatocytes. J Proteome Res.

[41] Connolly KD, Guschina IA, Yeung V, Clayton A, Draman MS, Von Ruhland C (2015). Characterisation of adipocyte-derived extracellular vesicles released pre- and post-adipogenesis. J Extracell Vesicles.

[42] Grange C, Tapparo M, Collino F, Vitillo L, Damasco C, Deregibus MC (2011). Microvesicles released from human renal cancer stem cells stimulate angiogenesis and formation of lung premetastatic niche. Can Res.

[43] Street JM, Birkhoff W, Menzies RI, Webb DJ, Bailey MA, Dear JW (2011). Exosomal transmission of functional aquaporin 2 in kidney cortical collecting duct cells. J Physiol.

[44] Gibaldi M, Perrier D. Pharmacokinetics. New York: M. Dekker; 1975. xi, 329 p. p.

[45] Caradec J, Kharmate G, Hosseini-Beheshti E, Adomat H, Gleave M, Guns E (2014). Reproducibility and efficiency of serum-derived exosome extraction methods. Clin Biochem.

[46] Chevillet JR, Kang Q, Ruf IK, Briggs HA, Vojtech LN, Hughes SM (2014). Quantitative and stoichiometric analysis of the microRNA content of exosomes. Proc Natl Acad Sci U S A.

[47] Papadopoulou C, Patrinou-Georgoula M, Guialis A (2010). Extensive association of HuR with hnRNP proteins within immunoselected hnRNP and mRNP complexes. Biochim Biophys Acta.

[48] Yuan F, Li Y-M, Wang Z (2021). Preserving extracellular vesicles for biomedical applications: consideration of storage stability before and after isolation. Drug Delivery.

[49] Wu J-Y, Li Y-J, Hu X-B, Huang S, Xiang D-X (2021). Preservation of small extracellular vesicles for functional analysis and therapeutic applications: a comparative evaluation of storage conditions. Drug Delivery.

[50] Mitchell PS, Parkin RK, Kroh EM, Fritz BR, Wyman SK, Pogosova-Agadjanyan EL (2008). Circulating microRNAs as stable blood-based markers for cancer detection. Proc Natl Acad Sci U S A.

[51] Li Y, Breaker RR (1999). Kinetics of RNA degradation by specific base catalysis of transesterification involving the 2‘-Hydroxyl group. J Am Chem Soc.

[52] Achour B, Al-Majdoub ZM, Grybos-Gajniak A, Lea K, Kilford P, Zhang M (2021). Liquid biopsy enables quantification of the abundance and interindividual variability of hepatic enzymes and transporters. Clin Pharmacol Ther.

[53] Rowland A, Ruanglertboon W, van Dyk M, Wijayakumara D, Wood LS, Meech R (2019). Plasma extracellular nanovesicle (exosome)-derived biomarkers for drug metabolism pathways: a novel approach to characterize variability in drug exposure. Br J Clin Pharmacol.

